# 1-(4-Chloro­butano­yl)-3-(2-nitro­phen­yl)thio­urea

**DOI:** 10.1107/S160053681202168X

**Published:** 2012-05-19

**Authors:** Nurziana Ngah, Maisara Kadir, Bohari M. Yamin, M. Sukeri M. Yusof

**Affiliations:** a Kulliyyah of Science, International Islamic University Malaysia, Bandar Indera Mahkota, 25200 Kuantan, Pahang, Malaysia; b Department of Chemical Sciences, Faculty of Science and Technology, Universiti Malaysia Terengganu, Menggabang Telipot, 21030 Kuala Terengganu, Malaysia; c School of Chemical Sciences and Food Technology, Faculty of Science and Technology, Universiti Kebangsaan Malaysia, UKM 43600 Bangi Selangor, Malaysia

## Abstract

The asymmetric unit of the title compound, C_11_H_12_ClN_3_O_3_S, contains two independent mol­ecules with different conformations in which the benzene ring and the thio­urea fragment form dihedral angles of 87.28 (12) and 66.44 (10)°. The O atom of the thio­amide group is involved in bifurcated N—H⋯O intra- and inter­molecular hydrogen bonding; the latter inter­action links the independent mol­ecules into a dimer. In the crystal, N—H⋯S inter­actions link the mol­ecules into chains propagating along the *c* axis.

## Related literature

For related structures, see: Yusof *et al.* (2011 ▶, 2012) ▶. For bond-length data, see: Allen *et al.* (1987 ▶).
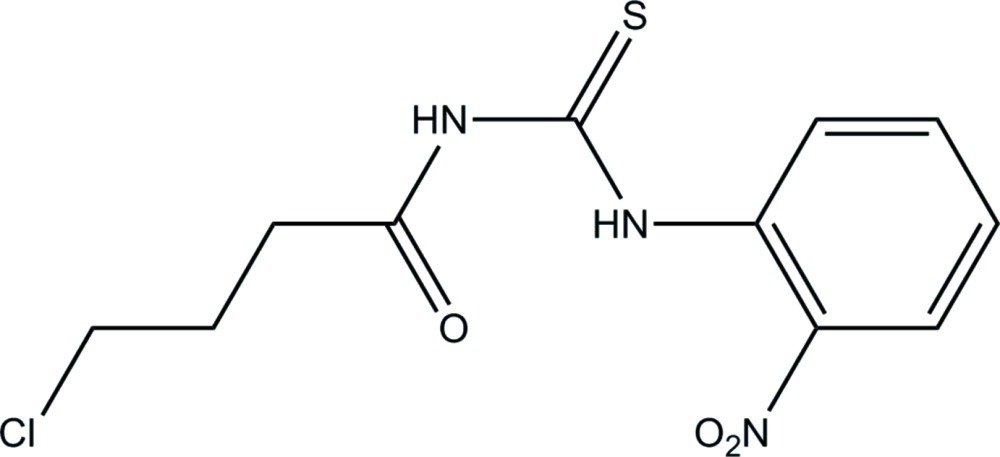


## Experimental

### 

#### Crystal data

C_11_H_12_ClN_3_O_3_S
*M* *_r_* = 301.75
Monoclinic, 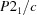

*a* = 14.593 (4) Å
*b* = 11.288 (3) Å
*c* = 17.828 (5) Å
β = 110.765 (5)°
*V* = 2745.8 (12) Å^3^
*Z* = 8
Mo *K*α radiation
μ = 0.44 mm^−1^
*T* = 298 K
0.36 × 0.35 × 0.34 mm

#### Data collection

Bruker SMART APEX CCD area-detector diffractometer
Absorption correction: multi-scan (*SADABS*; Bruker, 2000 ▶) *T*_min_ = 0.859, *T*_max_ = 0.866
15815 measured reflections
5117 independent reflections
3968 reflections with *I* > 2/s(*I*)
*R*_int_ = 0.028

#### Refinement

*R*[*F* ^2^ > 2σ(*F* ^2^)] = 0.046
*wR*(*F* ^2^) = 0.133
*S* = 1.03
5117 reflections
343 parameters
18 restraints
H-atom parameters constrained
Δρ_max_ = 0.73 e Å^−3^
Δρ_min_ = −0.44 e Å^−3^

### 

Data collection: *SMART* (Bruker, 2000 ▶); cell refinement: *SAINT* (Bruker, 2000 ▶); data reduction: *SAINT*; program(s) used to solve structure: *SHELXS97* (Sheldrick, 2008 ▶); program(s) used to refine structure: *SHELXL97* (Sheldrick, 2008 ▶); molecular graphics: *SHELXTL* (Sheldrick, 2008 ▶); software used to prepare material for publication: *SHELXTL*, *PARST* (Nardelli, 1995 ▶) and *PLATON* (Spek, 2009 ▶).

## Supplementary Material

Crystal structure: contains datablock(s) global, I. DOI: 10.1107/S160053681202168X/bq2354sup1.cif

Structure factors: contains datablock(s) I. DOI: 10.1107/S160053681202168X/bq2354Isup2.hkl

Supplementary material file. DOI: 10.1107/S160053681202168X/bq2354Isup3.cml

Additional supplementary materials:  crystallographic information; 3D view; checkCIF report

## Figures and Tables

**Table 1:** Hydrogen-bond geometry (Å, °)

*D*—H⋯*A*	*D*—H	H⋯*A*	*D*⋯*A*	*D*—H⋯*A*
N1—H1*A*⋯S2^i^	0.86	2.60	3.455 (2)	175
N2—H2*A*⋯O1	0.86	1.98	2.647 (3)	134
N2—H2*A*⋯O4	0.86	2.47	3.192 (3)	141
N4—H4*A*⋯S1^ii^	0.86	2.62	3.425 (2)	156
N5—H5*A*⋯O1	0.86	2.47	3.178 (3)	139
N5—H5*A*⋯O4	0.86	1.98	2.658 (3)	135

Symmetry codes: (i) 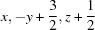; (ii) 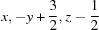.

**Fig. 1.**
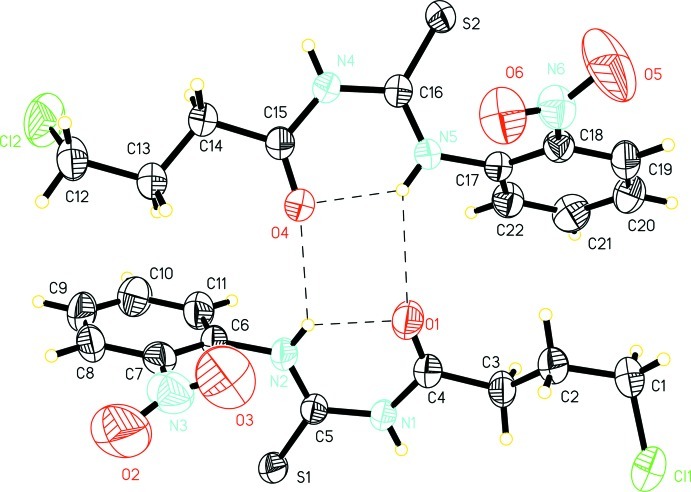
The molecular structure of (I), with 50% probability displacement ellipsoids.

**Fig. 2.**
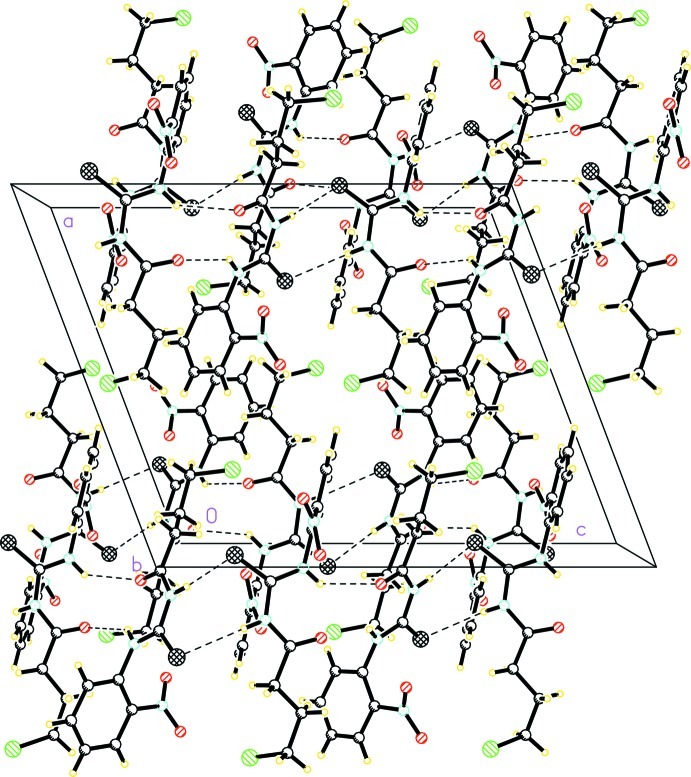
The molecular packing of (I) viewed down the b axis.

## supplementary crystallographic information

### Comment

The asymmetric unit of the title compound, (I), contains two crystallographically independent molecules with different conformations. The title molecule is similar to the previously reported 1-(4-chlorobutanoyl)-3-(2-chlorophenyl)thiourea (Yusof *et al.*, 2012) except the present of nitro group at the same position. The bond lengths and angles are in normal ranges (Allen *et al.*, 1987) and comparable with other similar substituent thiourea (Yusof *et al.*, 2011). The benzene rings [(C6—C11) & (C17—C22)] and thiourea fragments [(N1/N2/C4/S1/C5) & (N4/N5/C15/S2/C16)] are each planar with N5 atom deviates by 0.033 (2) Å from that plane. In each independent molecule, the benzene and thiourea fragments make dihedral angles of 87.28 (12)° and 66.44 (10)°, respectively and comparable to those reported by Yusof *et al.*, (2012). Each of oxygen atom in respective thioamide group [(C4/C5/O1/N1/N2/S1) & (C15/C16/O4/N4/N5/S2)] is involves in bifurcated intra and intermolecular N—H···O hydrogen bonds. The latter ones links the molecule into dimer. In the crystal structure, the molecule is further stabilized by N—H···S interactions to form one dimensional chain propagates along *c* axis.

### Experimental

30 ml acetone solution of 2-nitroanaline (1.83 g, 13.26 mmol) was added into a round-bottom flask containing a solution of 4-chlorobenzoylchloride (1.87 g, 13.26 mmol) and ammonium thiocyanate (1.00 g, 13.26 mmol). The solution mixture was refluxed for 1.5 h then filtered off and left to evaporate at room temperature. The yellowish precipitate obtained was washed with water and cold ethanol. The yellowish crystals were obtained by recrystallization of the precipitate in DMSO, suitable for X-ray analysis.

### Refinement

All H atoms were positioned geometrically and refined using riding model with C—H = 0.93 Å and N—H = 0.86 Å with *U*_iso_(H) = 1.2_eq_(C & N). A rigid body restraint (DELU in *SHELXL97*; Sheldrick, 2008) was applied to N3, N6, O2, O3, O5 and O6 atoms. The ISOR (*SHELXTL97*; Sheldrick, 2008) was applied to O2 and O5 atoms.

### Crystal data

**Table:**

C_11_H_12_ClN_3_O_3_S	*Z* = 8
*M**_r_* = 301.75	*F*(000) = 1248
Monoclinic, *P*2_1_/*c*	*D*_x_ = 1.460 Mg m^−^^3^
Hall symbol: -P 2ybc	Mo *K*α radiation, λ = 0.71073 Å
*a* = 14.593 (4) Å	θ = 2.1–25.5°
*b* = 11.288 (3) Å	µ = 0.44 mm^−^^1^
*c* = 17.828 (5) Å	*T* = 298 K
β = 110.765 (5)°	Block, light yellow
*V* = 2745.8 (12) Å^3^	0.36 × 0.35 × 0.34 mm

### Data collection

**Table:**

Bruker SMART APEX CCD area-detector diffractometer	5117 independent reflections
Radiation source: fine-focus sealed tube	3968 reflections with *I* > 2/s(*I*)
Graphite monochromator	*R*_int_ = 0.028
Detector resolution: 83.66 pixels mm^-1^	θ_max_ = 25.5°, θ_min_ = 2.1°
ω scan	*h* = −17→17
Absorption correction: multi-scan (*SADABS*; Bruker, 2000)	*k* = −13→13
*T*_min_ = 0.859, *T*_max_ = 0.866	*l* = −21→15
15815 measured reflections	

### Refinement

**Table:**

Refinement on *F*^2^	Primary atom site location: structure-invariant direct methods
Least-squares matrix: full	Secondary atom site location: difference Fourier map
*R*[*F*^2^ > 2σ(*F*^2^)] = 0.046	Hydrogen site location: inferred from neighbouring sites
*wR*(*F*^2^) = 0.133	H-atom parameters constrained
*S* = 1.03	*w* = 1/[σ^2^(*F*_o_^2^) + (0.0667*P*)^2^ + 1.5901*P*] where *P* = (*F*_o_^2^ + 2*F*_c_^2^)/3
5117 reflections	(Δ/σ)_max_ < 0.001
343 parameters	Δρ_max_ = 0.73 e Å^−^^3^
18 restraints	Δρ_min_ = −0.44 e Å^−^^3^

### Special details

**Table:**

Geometry. All e.s.d.'s (except the e.s.d. in the dihedral angle between two l.s. planes) are estimated using the full covariance matrix. The cell e.s.d.'s are taken into account individually in the estimation of e.s.d.'s in distances, angles and torsion angles; correlations between e.s.d.'s in cell parameters are only used when they are defined by crystal symmetry. An approximate (isotropic) treatment of cell e.s.d.'s is used for estimating e.s.d.'s involving l.s. planes.
Refinement. Refinement of *F*^2^ against ALL reflections. The weighted *R*-factor *wR* and goodness of fit *S* are based on *F*^2^, conventional *R*-factors *R* are based on *F*, with *F* set to zero for negative *F*^2^. The threshold expression of *F*^2^ > σ(*F*^2^) is used only for calculating *R*-factors(gt) *etc*. and is not relevant to the choice of reflections for refinement. *R*-factors based on *F*^2^ are statistically about twice as large as those based on *F*, and *R*- factors based on ALL data will be even larger.

### Fractional atomic coordinates and isotropic or equivalent isotropic displacement parameters (Å^2^)

**Table:**

	*x*	*y*	*z*	*U*_iso_*/*U*_eq_	
Cl1	1.24412 (6)	0.79386 (9)	0.71487 (5)	0.0756 (3)	
Cl2	0.47919 (7)	0.83444 (9)	0.03285 (6)	0.0889 (3)	
S1	0.76404 (5)	0.50753 (6)	0.47256 (4)	0.0493 (2)	
S2	1.03228 (4)	1.01443 (6)	0.16703 (4)	0.04415 (18)	
O1	0.94192 (14)	0.81777 (18)	0.43547 (12)	0.0606 (6)	
O2	0.5292 (2)	0.8151 (3)	0.3985 (2)	0.1216 (12)	
O3	0.6705 (3)	0.8670 (3)	0.4071 (2)	0.1155 (11)	
O4	0.80541 (12)	0.89874 (19)	0.27305 (11)	0.0522 (5)	
O5	1.1880 (2)	1.2063 (2)	0.3510 (3)	0.1318 (15)	
O6	1.04315 (17)	1.16326 (19)	0.34087 (16)	0.0728 (6)	
N1	0.91463 (14)	0.65268 (19)	0.49776 (12)	0.0413 (5)	
H1A	0.9404	0.6088	0.5394	0.050*	
N2	0.78153 (14)	0.6862 (2)	0.38280 (13)	0.0455 (5)	
H2A	0.8131	0.7465	0.3750	0.055*	
N3	0.6020 (2)	0.8010 (3)	0.38258 (19)	0.0722 (8)	
N4	0.85978 (13)	0.97251 (17)	0.17751 (12)	0.0364 (4)	
H4A	0.8398	1.0006	0.1296	0.044*	
N5	0.99262 (13)	0.93177 (18)	0.29187 (12)	0.0386 (5)	
H5A	0.9500	0.9139	0.3132	0.046*	
N6	1.12681 (18)	1.1360 (2)	0.35275 (16)	0.0560 (6)	
C1	1.2274 (2)	0.8592 (3)	0.61931 (19)	0.0652 (9)	
H1B	1.2546	0.9385	0.6274	0.078*	
H1C	1.2628	0.8130	0.5926	0.078*	
C2	1.12111 (19)	0.8653 (3)	0.56658 (17)	0.0510 (7)	
H2B	1.1155	0.9037	0.5165	0.061*	
H2C	1.0861	0.9132	0.5927	0.061*	
C3	1.07414 (17)	0.7445 (2)	0.54881 (16)	0.0448 (6)	
H3A	1.1143	0.6938	0.5290	0.054*	
H3B	1.0731	0.7103	0.5984	0.054*	
C4	0.97212 (17)	0.7454 (2)	0.48887 (15)	0.0413 (6)	
C5	0.82146 (17)	0.6218 (2)	0.44845 (14)	0.0382 (5)	
C6	0.68819 (17)	0.6588 (2)	0.32493 (15)	0.0426 (6)	
C7	0.60209 (19)	0.7115 (3)	0.32442 (17)	0.0494 (7)	
C8	0.5123 (2)	0.6819 (3)	0.2662 (2)	0.0657 (9)	
H8A	0.4548	0.7167	0.2667	0.079*	
C9	0.5099 (2)	0.6008 (4)	0.2084 (2)	0.0735 (10)	
H9A	0.4504	0.5816	0.1688	0.088*	
C10	0.5934 (2)	0.5483 (3)	0.2086 (2)	0.0709 (9)	
H10A	0.5907	0.4929	0.1693	0.085*	
C11	0.6824 (2)	0.5763 (3)	0.26647 (17)	0.0572 (7)	
H11A	0.7391	0.5391	0.2660	0.069*	
C12	0.5051 (2)	0.9274 (3)	0.11797 (19)	0.0635 (8)	
H12A	0.4964	1.0095	0.1007	0.076*	
H12B	0.4592	0.9105	0.1448	0.076*	
C13	0.60803 (18)	0.9100 (3)	0.17614 (16)	0.0526 (7)	
H13A	0.6158	0.9539	0.2248	0.063*	
H13B	0.6175	0.8267	0.1902	0.063*	
C14	0.68560 (17)	0.9485 (2)	0.14479 (15)	0.0441 (6)	
H14A	0.6744	1.0307	0.1282	0.053*	
H14B	0.6804	0.9016	0.0979	0.053*	
C15	0.78730 (17)	0.9361 (2)	0.20533 (15)	0.0387 (5)	
C16	0.96013 (16)	0.9697 (2)	0.21656 (14)	0.0349 (5)	
C17	1.09327 (16)	0.9188 (2)	0.33935 (14)	0.0358 (5)	
C18	1.15787 (17)	1.0128 (2)	0.36830 (15)	0.0384 (5)	
C19	1.25516 (19)	0.9937 (3)	0.41403 (17)	0.0500 (7)	
H19A	1.2975	1.0576	0.4321	0.060*	
C20	1.2893 (2)	0.8798 (3)	0.43287 (18)	0.0557 (7)	
H20A	1.3547	0.8664	0.4638	0.067*	
C21	1.2264 (2)	0.7864 (3)	0.40572 (18)	0.0564 (7)	
H21A	1.2492	0.7095	0.4189	0.068*	
C22	1.12941 (19)	0.8053 (2)	0.35899 (16)	0.0464 (6)	
H22A	1.0879	0.7408	0.3405	0.056*	

### Atomic displacement parameters (Å^2^)

**Table:**

	*U*^11^	*U*^22^	*U*^33^	*U*^12^	*U*^13^	*U*^23^
Cl1	0.0570 (5)	0.0986 (7)	0.0547 (5)	−0.0123 (4)	−0.0006 (4)	−0.0010 (4)
Cl2	0.0714 (6)	0.0930 (7)	0.0774 (6)	−0.0241 (5)	−0.0043 (5)	−0.0185 (5)
S1	0.0428 (4)	0.0587 (4)	0.0406 (4)	−0.0163 (3)	0.0077 (3)	0.0052 (3)
S2	0.0353 (3)	0.0575 (4)	0.0400 (3)	−0.0052 (3)	0.0138 (3)	0.0014 (3)
O1	0.0468 (11)	0.0608 (12)	0.0579 (12)	−0.0145 (9)	−0.0014 (9)	0.0186 (10)
O2	0.111 (2)	0.158 (3)	0.120 (3)	0.053 (2)	0.072 (2)	0.018 (2)
O3	0.124 (3)	0.106 (2)	0.126 (3)	−0.019 (2)	0.056 (2)	−0.044 (2)
O4	0.0342 (9)	0.0766 (14)	0.0419 (10)	−0.0042 (9)	0.0085 (8)	0.0142 (9)
O5	0.0838 (19)	0.0484 (14)	0.253 (5)	−0.0123 (14)	0.047 (2)	0.031 (2)
O6	0.0634 (14)	0.0513 (12)	0.1023 (18)	0.0153 (10)	0.0277 (13)	−0.0092 (12)
N1	0.0333 (10)	0.0483 (12)	0.0363 (11)	−0.0070 (9)	0.0048 (8)	0.0053 (9)
N2	0.0338 (11)	0.0521 (13)	0.0433 (12)	−0.0100 (9)	0.0046 (9)	0.0086 (10)
N3	0.0732 (19)	0.0735 (19)	0.079 (2)	0.0116 (15)	0.0388 (17)	0.0104 (15)
N4	0.0310 (10)	0.0401 (11)	0.0347 (10)	0.0000 (8)	0.0074 (8)	0.0044 (8)
N5	0.0280 (10)	0.0481 (12)	0.0373 (11)	−0.0027 (8)	0.0088 (8)	0.0055 (9)
N6	0.0536 (15)	0.0385 (12)	0.0713 (16)	−0.0029 (11)	0.0163 (12)	−0.0024 (11)
C1	0.0432 (16)	0.090 (2)	0.0577 (18)	−0.0242 (15)	0.0115 (14)	−0.0058 (16)
C2	0.0436 (15)	0.0568 (17)	0.0479 (15)	−0.0148 (12)	0.0104 (12)	−0.0011 (13)
C3	0.0315 (12)	0.0504 (15)	0.0471 (14)	−0.0041 (11)	0.0074 (11)	−0.0006 (12)
C4	0.0355 (13)	0.0455 (14)	0.0397 (13)	−0.0043 (11)	0.0094 (11)	−0.0023 (11)
C5	0.0321 (12)	0.0464 (14)	0.0340 (12)	−0.0016 (10)	0.0091 (10)	−0.0034 (10)
C6	0.0308 (12)	0.0524 (15)	0.0394 (13)	−0.0067 (11)	0.0061 (10)	0.0086 (11)
C7	0.0424 (14)	0.0545 (16)	0.0523 (16)	0.0018 (12)	0.0181 (12)	0.0155 (13)
C8	0.0303 (14)	0.088 (2)	0.074 (2)	0.0007 (14)	0.0128 (14)	0.0335 (19)
C9	0.0416 (17)	0.101 (3)	0.060 (2)	−0.0244 (17)	−0.0032 (14)	0.0148 (19)
C10	0.058 (2)	0.090 (2)	0.0549 (18)	−0.0229 (18)	0.0081 (15)	−0.0102 (17)
C11	0.0424 (15)	0.071 (2)	0.0540 (17)	−0.0072 (14)	0.0116 (13)	−0.0057 (15)
C12	0.0357 (14)	0.091 (2)	0.0580 (18)	−0.0044 (15)	0.0101 (13)	0.0002 (17)
C13	0.0335 (13)	0.076 (2)	0.0444 (15)	−0.0032 (13)	0.0094 (11)	0.0044 (13)
C14	0.0332 (12)	0.0538 (15)	0.0413 (14)	−0.0008 (11)	0.0084 (10)	0.0074 (12)
C15	0.0327 (12)	0.0411 (13)	0.0395 (13)	−0.0021 (10)	0.0092 (10)	0.0021 (11)
C16	0.0322 (12)	0.0326 (12)	0.0372 (13)	0.0004 (9)	0.0089 (10)	−0.0031 (9)
C17	0.0315 (12)	0.0413 (13)	0.0336 (12)	−0.0008 (10)	0.0105 (9)	0.0022 (10)
C18	0.0364 (12)	0.0370 (13)	0.0403 (13)	−0.0001 (10)	0.0116 (10)	0.0018 (10)
C19	0.0373 (13)	0.0549 (17)	0.0518 (16)	−0.0089 (12)	0.0082 (12)	−0.0027 (13)
C20	0.0358 (14)	0.0668 (19)	0.0558 (17)	0.0091 (13)	0.0056 (12)	0.0111 (14)
C21	0.0533 (17)	0.0462 (16)	0.0656 (18)	0.0154 (13)	0.0159 (14)	0.0118 (14)
C22	0.0442 (14)	0.0384 (14)	0.0521 (15)	−0.0009 (11)	0.0115 (12)	0.0001 (11)

### Geometric parameters (Å, º)

**Table:**

Cl1—C1	1.792 (3)	C3—H3A	0.9700
Cl2—C12	1.772 (3)	C3—H3B	0.9700
S1—C5	1.675 (3)	C6—C11	1.378 (4)
S2—C16	1.674 (2)	C6—C7	1.387 (4)
O1—C4	1.213 (3)	C7—C8	1.393 (4)
O2—N3	1.205 (4)	C8—C9	1.369 (5)
O3—N3	1.198 (4)	C8—H8A	0.9300
O4—C15	1.216 (3)	C9—C10	1.354 (5)
O5—N6	1.203 (3)	C9—H9A	0.9300
O6—N6	1.203 (3)	C10—C11	1.379 (4)
N1—C5	1.377 (3)	C10—H10A	0.9300
N1—C4	1.385 (3)	C11—H11A	0.9300
N1—H1A	0.8600	C12—C13	1.505 (4)
N2—C5	1.324 (3)	C12—H12A	0.9700
N2—C6	1.421 (3)	C12—H12B	0.9700
N2—H2A	0.8600	C13—C14	1.494 (4)
N3—C7	1.447 (4)	C13—H13A	0.9700
N4—C15	1.380 (3)	C13—H13B	0.9700
N4—C16	1.381 (3)	C14—C15	1.500 (3)
N4—H4A	0.8600	C14—H14A	0.9700
N5—C16	1.326 (3)	C14—H14B	0.9700
N5—C17	1.419 (3)	C17—C22	1.382 (3)
N5—H5A	0.8600	C17—C18	1.391 (3)
N6—C18	1.458 (3)	C18—C19	1.381 (4)
C1—C2	1.503 (4)	C19—C20	1.376 (4)
C1—H1B	0.9700	C19—H19A	0.9300
C1—H1C	0.9700	C20—C21	1.369 (4)
C2—C3	1.508 (4)	C20—H20A	0.9300
C2—H2B	0.9700	C21—C22	1.380 (4)
C2—H2C	0.9700	C21—H21A	0.9300
C3—C4	1.493 (3)	C22—H22A	0.9300
			
C5—N1—C4	128.3 (2)	C10—C9—C8	120.5 (3)
C5—N1—H1A	115.9	C10—C9—H9A	119.7
C4—N1—H1A	115.9	C8—C9—H9A	119.7
C5—N2—C6	122.0 (2)	C9—C10—C11	120.6 (3)
C5—N2—H2A	119.0	C9—C10—H10A	119.7
C6—N2—H2A	119.0	C11—C10—H10A	119.7
O3—N3—O2	121.6 (4)	C6—C11—C10	120.7 (3)
O3—N3—C7	118.9 (3)	C6—C11—H11A	119.7
O2—N3—C7	119.1 (3)	C10—C11—H11A	119.7
C15—N4—C16	128.5 (2)	C13—C12—Cl2	111.7 (2)
C15—N4—H4A	115.7	C13—C12—H12A	109.3
C16—N4—H4A	115.7	Cl2—C12—H12A	109.3
C16—N5—C17	124.1 (2)	C13—C12—H12B	109.3
C16—N5—H5A	118.0	Cl2—C12—H12B	109.3
C17—N5—H5A	118.0	H12A—C12—H12B	107.9
O6—N6—O5	122.9 (3)	C14—C13—C12	114.1 (2)
O6—N6—C18	120.3 (2)	C14—C13—H13A	108.7
O5—N6—C18	116.8 (3)	C12—C13—H13A	108.7
C2—C1—Cl1	112.1 (2)	C14—C13—H13B	108.7
C2—C1—H1B	109.2	C12—C13—H13B	108.7
Cl1—C1—H1B	109.2	H13A—C13—H13B	107.6
C2—C1—H1C	109.2	C13—C14—C15	113.0 (2)
Cl1—C1—H1C	109.2	C13—C14—H14A	109.0
H1B—C1—H1C	107.9	C15—C14—H14A	109.0
C1—C2—C3	112.4 (2)	C13—C14—H14B	109.0
C1—C2—H2B	109.1	C15—C14—H14B	109.0
C3—C2—H2B	109.1	H14A—C14—H14B	107.8
C1—C2—H2C	109.1	O4—C15—N4	122.4 (2)
C3—C2—H2C	109.1	O4—C15—C14	123.9 (2)
H2B—C2—H2C	107.9	N4—C15—C14	113.7 (2)
C4—C3—C2	114.1 (2)	N5—C16—N4	116.9 (2)
C4—C3—H3A	108.7	N5—C16—S2	124.43 (18)
C2—C3—H3A	108.7	N4—C16—S2	118.69 (17)
C4—C3—H3B	108.7	C22—C17—C18	117.7 (2)
C2—C3—H3B	108.7	C22—C17—N5	117.9 (2)
H3A—C3—H3B	107.6	C18—C17—N5	124.4 (2)
O1—C4—N1	122.2 (2)	C19—C18—C17	121.3 (2)
O1—C4—C3	124.0 (2)	C19—C18—N6	116.5 (2)
N1—C4—C3	113.8 (2)	C17—C18—N6	122.2 (2)
N2—C5—N1	116.8 (2)	C20—C19—C18	119.8 (2)
N2—C5—S1	123.08 (18)	C20—C19—H19A	120.1
N1—C5—S1	120.11 (18)	C18—C19—H19A	120.1
C11—C6—C7	118.2 (2)	C21—C20—C19	119.6 (2)
C11—C6—N2	118.8 (2)	C21—C20—H20A	120.2
C7—C6—N2	123.0 (3)	C19—C20—H20A	120.2
C6—C7—C8	120.8 (3)	C20—C21—C22	120.7 (3)
C6—C7—N3	121.7 (3)	C20—C21—H21A	119.7
C8—C7—N3	117.5 (3)	C22—C21—H21A	119.7
C9—C8—C7	119.2 (3)	C21—C22—C17	120.9 (2)
C9—C8—H8A	120.4	C21—C22—H22A	119.6
C7—C8—H8A	120.4	C17—C22—H22A	119.6
			
Cl1—C1—C2—C3	−60.9 (3)	Cl2—C12—C13—C14	66.4 (3)
C1—C2—C3—C4	−173.1 (2)	C12—C13—C14—C15	177.0 (3)
C5—N1—C4—O1	4.6 (4)	C16—N4—C15—O4	2.7 (4)
C5—N1—C4—C3	−173.4 (2)	C16—N4—C15—C14	−177.9 (2)
C2—C3—C4—O1	29.3 (4)	C13—C14—C15—O4	0.0 (4)
C2—C3—C4—N1	−152.8 (2)	C13—C14—C15—N4	−179.4 (2)
C6—N2—C5—N1	176.2 (2)	C17—N5—C16—N4	177.3 (2)
C6—N2—C5—S1	−3.7 (4)	C17—N5—C16—S2	−3.1 (3)
C4—N1—C5—N2	1.9 (4)	C15—N4—C16—N5	−2.9 (4)
C4—N1—C5—S1	−178.2 (2)	C15—N4—C16—S2	177.4 (2)
C5—N2—C6—C11	−84.7 (3)	C16—N5—C17—C22	−110.9 (3)
C5—N2—C6—C7	95.3 (3)	C16—N5—C17—C18	69.9 (3)
C11—C6—C7—C8	−0.1 (4)	C22—C17—C18—C19	1.0 (4)
N2—C6—C7—C8	179.8 (2)	N5—C17—C18—C19	−179.8 (2)
C11—C6—C7—N3	−178.6 (3)	C22—C17—C18—N6	−178.5 (2)
N2—C6—C7—N3	1.4 (4)	N5—C17—C18—N6	0.7 (4)
O3—N3—C7—C6	32.9 (5)	O6—N6—C18—C19	−152.6 (3)
O2—N3—C7—C6	−154.8 (3)	O5—N6—C18—C19	28.9 (4)
O3—N3—C7—C8	−145.6 (4)	O6—N6—C18—C17	27.0 (4)
O2—N3—C7—C8	26.7 (4)	O5—N6—C18—C17	−151.6 (3)
C6—C7—C8—C9	−0.9 (4)	C17—C18—C19—C20	−1.1 (4)
N3—C7—C8—C9	177.6 (3)	N6—C18—C19—C20	178.4 (3)
C7—C8—C9—C10	1.2 (5)	C18—C19—C20—C21	0.3 (4)
C8—C9—C10—C11	−0.6 (5)	C19—C20—C21—C22	0.7 (5)
C7—C6—C11—C10	0.8 (4)	C20—C21—C22—C17	−0.8 (4)
N2—C6—C11—C10	−179.2 (3)	C18—C17—C22—C21	0.0 (4)
C9—C10—C11—C6	−0.5 (5)	N5—C17—C22—C21	−179.3 (2)

### Hydrogen-bond geometry (Å, º)

**Table:**

*D*—H···*A*	*D*—H	H···*A*	*D*···*A*	*D*—H···*A*
N2—H2*A*···O1	0.86	1.98	2.647 (3)	134
N5—H5*A*···O4	0.86	1.98	2.658 (3)	135
N2—H2*A*···O4	0.86	2.47	3.192 (3)	141
N5—H5*A*···O1	0.86	2.47	3.178 (3)	139
N1—H1*A*···S2^i^	0.86	2.60	3.455 (2)	175
N4—H4*A*···S1^ii^	0.86	2.62	3.425 (2)	156

Symmetry codes: (i) *x*, −*y*+3/2, *z*+1/2; (ii) *x*, −*y*+3/2, *z*−1/2.
